# Calcitriol Suppresses HIF-1 and HIF-2 Transcriptional Activity by Reducing HIF-1/2α Protein Levels via a VDR-Independent Mechanism

**DOI:** 10.3390/cells9112440

**Published:** 2020-11-09

**Authors:** Ioanna-Maria Gkotinakou, Eleni Kechagia, Kalliopi Pazaitou-Panayiotou, Ilias Mylonis, Panagiotis Liakos, Andreas Tsakalof

**Affiliations:** 1 Laboratory of Biochemistry, Faculty of Medicine, University of Thessaly, Biopolis 41500, Larissa, Greece; iogotina@med.uth.gr (I.-M.G.); elkechag@uth.gr (E.K.); pliakos@med.uth.gr (P.L.); 2 Division of Endocrinology, Interbalkan Medical Center, Thessaloniki 55535, Greece; kpazaitoupanayiotou@gmail.com

**Keywords:** vitamin D, calcitriol, hypoxia, hypoxia-inducible factors, VDR, cancer

## Abstract

Hypoxia-inducible transcription factors 1 and 2 (HIFs) are major mediators of cancer development and progression and validated targets for cancer therapy. Although calcitriol, the biologically active metabolite of vitamin D, was attributed with anticancer properties, there is little information on the effect of calcitriol on HIFs and the mechanism underling this activity. Here, we demonstrate the negative effect of calcitriol on HIF-1/2α protein levels and HIF-1/2 transcriptional activity and elucidate the molecular mechanism of calcitriol action. We also reveal that the suppression of vitamin D receptor (VDR) expression by siRNA does not abrogate the negative regulation of HIF-1α and HIF-2α protein levels and HIF-1/2 transcriptional activity by calcitriol, thus testifying that the mechanism of these actions is VDR independent. At the same time, calcitriol significantly reduces the phosphorylation of Akt protein kinase and its downstream targets and suppresses HIF-1/2α protein synthesis by inhibiting *HIF1A* and *EPAS1* (Endothelial PAS domain-containing protein 1) mRNA translation, without affecting their mRNA levels. On the basis of the acquired data, it can be proposed that calcitriol reduces HIF-1α and HIF-2α protein levels and inhibits HIF-1 and HIF-2 transcriptional activity by a VDR-independent, nongenomic mechanism that involves inhibition of PI3K/Akt signaling pathway and suppression of *HIF1A* and *EPAS1* mRNA translation.

## 1. Introduction

Vitamin D belongs to a group of fat-soluble secosteroids and is primarily responsible for bone development and calcium homeostasis. At the same time, epidemiological studies indicate that vitamin D deficiency can play an etiological role in the development of various human cancers [1] and other chronic diseases. Moreover, a plethora of cell-based studies support that vitamin D has strong and diverse anticancer potential via calcitriol (1α,25-dihydroxyvitamin D or 1,25(OH)_2_D_3_), its metabolically active hormonal form [2,3]. However, clinical exploitation of this potential for cancer prevention and treatment is still debatable, and available clinical trials lead to inconsistent and controversial conclusions, probably due to the poor design of clinical trials [4,5,6] that frequently do not take under consideration the difference between nutrients and drugs [6] as well as the whole complexity of vitamin D metabolic pathway and mode of action [7].

Calcitriol, like other steroids, exerts its effects through genomic and nongenomic mechanisms [8,9]. The genomic mechanism occurs via vitamin D receptor (VDR), a member of the steroid–thyroid–retinoid receptor superfamily of ligand-activated transcription factors. Binding of calcitriol to VDR promotes heterodimerization with retinoid X receptor (RXR) and subsequent binding of calcitriol-VDR-RXR complex to VDREs (vitamin D-response elements) in DNA to initiate gene transcription. Transcriptional responses to calcitriol generated mainly by activation of the VDR nuclear receptor or, in some cases, independently of VDR by influencing factors like Sp-1 (specificity protein 1) or MZF-1 (myeloid zinc finger-1) [10,11] generally take several hours to days. Since some cellular responses to calcitriol occur too quickly to be mediated by transcriptional events, evidence for membrane-associated receptors has been reported. Recent data suggest that nongenomic calcitriol functions are mediated by the activation of a membrane-associated rapid response steroid-binding (MARRS) receptor (also known as ERp57/GRp58) and induce rapid cellular response [8,12]. MARRS-activated signaling triggers a number of signaling cascades that are crucial for cancer development and progression, such as the PI3K/Akt and MAPK signaling pathways [2,13,14,15].

The PI3K/Akt signaling is perhaps one of the most commonly activated pathways in human cancer that is implicated primarily in the synthesis of proteins that control/regulate vital cellular processes of cancer cell survival, proliferation, and metabolism [16]. The hypoxia-inducible factors 1α (HIF-1α) and 2α (HIF-2α) are among these proteins [17,18]. HIFs, the master regulators of cellular response to hypoxia, constitute a family of heterodimeric transcription factors composed of one oxygen labile subunit (HIF-1α, -2α or 3α) and a constitutively expressed β subunit HIF-1β (ARNT; aryl hydrocarbon receptor nuclear translocator) and are highly involved in the pathology of cancer and other diseases associated with tissue hypoxia (reduced oxygen tension) [19]. Under physiological oxygen tension, normoxia, oxygen-dependent prolyl hydroxylases (PHDs) modify HIF-α subunits and cause their Von Hippel-Lindau (pVHL)-mediated polyubiquitination and subsequent proteasomal degradation. Under reduced oxygen tension (hypoxia) PHDs are deactivated, subsequently leading to HIF-α subunit stabilization, its rapid accumulation, nuclear translocation, and dimerization with HIF-1β [20]. Binding of the resulting HIF complex to hypoxia-response elements (HREs) of target genes and recruitment of transcriptional coactivators induces genes expression that mediates cells response to hypoxia by promoting anaerobic metabolism, erythropoiesis, angiogenesis, and other cellular and systemic responses that ultimately enable cancer cells survival under hypoxia and metastasis [19].

In addition to oxygen-dependent control, the expression of HIF-α subunits, and HIF transcriptional activity can also be regulated in an oxygen-independent manner through major signal transduction pathways [21,22]. In particular, PI3K/Akt/mTOR pathway activation or inhibition has been specifically implicated in increased or reduced, correspondingly, protein synthesis of HIF-α subunits [17,23,24,25,26,27]. Although PI3K/Akt/mTOR pathway is usually linked with augmented HIF-α levels via translational control, Akt signaling influences processes such as GSK3 activity that is implicated with HIF-1α stability in a VHL-independent manner [28] or influence ROS-mediated HIF-α regulation [29]. The subsequent nuclear accumulation of HIFs-α and dimerization with HIF-β is controlled through HIF-1α and HIF-2α phosphorylation by ERK1/2 [30,31,32]. HIF-1 and HIF-2 are particularly important for hypoxic response, while the role of HIF-3 is investigated and understood to a lesser extent [24,33]. Although homologous, the different HIF isoforms possess shared but also distinct functions. Both HIF-1 and HIF-2 transcriptionally activate VEGF-A, a key contributor in the formation of new blood vessels. HIF-1 has a more prominent role in the regulation of genes encoding metabolic enzymes, such as (*PGK1*) [34]. Specific targets of HIF-2 include genes involved in physiological processes such as erythropoiesis (*EPO*) [35], which is recently found to be also regulated by a long HIF-3α isoform [36] and in pathological conditions including solid tumors progression and metastasis (*SERPINE1*, *MMP2*, and *TGFA*) [37,38,39]. HIFs activation is observed in many solid tumors and leads to the expression of genes that enable cancer cells’ adaptation and survival under hypoxic conditions [40,41].

In the present study, we investigated the effect of calcitriol on HIF-1α and HIF-2α protein expression and HIF-1 and 2 transcriptional activity using as a model the Huh7 human hepatocarcinoma cells. We studied the molecular mechanism of this effect and demonstrate that calcitriol suppresses HIF-1α and HIF-2α subunits protein synthesis implicates negative regulation of the PI3K/AKT/mTOR signaling pathway, through a VDR-independent, nongenomic mechanism.

Better understanding of vitamin D anticancer actions and molecular mechanisms could contribute to more efficient future clinical applications of vitamin in cancer treatment and prevention.

## 2. Materials and Methods

### 2.1. Cell Culture, Transfection, and Luciferase Assays

Huh7 and HepG2 cells were purchased from the European Collection of Cell Cultures (ECACC) and tested for mycoplasma. Cells were cultured in Dulbecco’s Modified Eagle’s Medium (DMEM; Biosera, France) containing 10% fetal bovine serum (FBS; Biochrom, Berlin, Germany) and 100 U/mL penicillin–streptomycin (Biosera, France) in normal atmospheric air containing 5% CO_2_ (denoted as 21% O_2_) in a standard humidified incubator. For hypoxic treatment, cells were exposed for 8 or 24 h to a gas mixture containing 1% O_2_, 94% N_2_, and 5% CO_2_ (denoted as 1% O_2_) in an IN VIVO2 200 hypoxia workstation (Baker Ruskinn, Sanford, ME, USA). When required, cells were pretreated for 1 h with 20, 50, or 100 nM of 1,25(OH)_2_D3 (Sigma-Aldrich, Darmstadt, Germany) in DMEM containing 5% FBS and 100 U/mL penicillin–streptomycin and grown for 8 or 24 h under 21% or 1% O_2_, respectively. For proteasome inhibition experiments, cells were treated with 100 nM of 1,25(OH)_2_D3 for 1 h and exposed to hypoxia (1% O_2_) for 8 or 24 h. For this, 4 h before lysis, cells were treated with 10 μΜ MG132 (10012628, Cayman, Ann Arbor, MI, USA). When needed, 1 mM of DMOG (dimethyloxallyl glycine; 71210, Cayman, Ann Arbor, MI, USA) was added as indicated. Transient transfections were carried out in 24-well plates using TurboFect™ Transfection Reagent (Thermo Fisher Scientific Inc., Waltham, MA, USA) according to the manufacturer’s instructions. When required, 24 h post-transfection, cells were pretreated for 1 h with 50 or 100 nM of 1,25(OH)_2_D3 in DMEM containing 5% FBS and 100 U/mL penicillin–streptomycin and exposed to 1% O_2_ for 8 or 24 h. Luciferase assays were performed in cells transiently cotransfected with the firefly luciferase reporter plasmid pGL3–5HRE-VEGF and the Renilla luciferase expressing plasmid PCI Renilla. Cells were washed with PBS, lysed, and luciferase activity was determined using the Dual Luciferase Reporter Assay System (Promega, Madison, WI, USA) with a luminometer (TD20/20, Turner Designs, San Jose, CA, USA).

### 2.2. siRNA-Mediated Silencing

Huh7 cells were incubated in DMEM medium with siRNA targeting VDR (10 nM; H00007421-R01, Abnova, Taipei, Taiwan) using Viromer^®^BLUE (Lipocalyx, Halle, Germany) as described by the manufacturer. AllStars siRNA (1027280, Qiagen, Venlo, The Netherlands) was used as negative control. Then, 48 h post-transfection, cells were pretreated for 1 h with 50 or 100 nM of 1,25(OH)_2_D3 in DMEM containing 5% FBS and 100 U/mL penicillin–streptomycin and exposed to 1% O_2_ for 8 or 24 h. Then the cells were harvested, and cell lysates were prepared for Western blot analysis, luciferase assay or RT-PCR.

### 2.3. SDS-PAGE and Western Blot

Proteins were resolved by 10% SDS-PAGE gels and analyzed by Western immunoblotting using the antibodies: rabbit polyclonal antibody against HIF-2α (ΝΒ100-122, 1:750 dilution; Novus Europe, Cambridge, UK), affinity purified rabbit polyclonal antibody against HIF-1α (1:1000 dilution, reported in [42]), mouse monoclonal antibody against ARNT (611079, 1:500; BD Biosciences, San Jose, CA, USA), mouse monoclonal antibody against β-actin (3700, 1:5000 dilution; Cell Signaling, Danvers, MA, USA), rabbit monoclonal antibody against phospho-Akt (Ser473) (4060, 1:1000 dilution; Cell Signaling), rabbit monoclonal antibody against Akt (pan) (4685, 1:1000 dilution; Cell Signaling), rabbit polyclonal antibodies against ERK1/2 and Phospho-ERK1/2 (9102 and 9101, respectively, 1:1000 dilution; Cell Signaling), rabbit monoclonal antibody against phospho-4E-BP1 (Thr37/46) (236B4) (2855, 1:1000 dilution; Cell Signaling), rabbit monoclonal antibody against phospho-eIF4E (Ser209) antibody (9741, 1:1000 dilution; Cell Signaling) mouse polyclonal antibody against VDR (H00007421-B01P, 1:500 dilution; Abnova), mouse monoclonal antibody against α-Tubulin (DM1A) (3873, 1:10000 dilution; Cell Signaling) and a rabbit polyclonal antibody against VEGF (sc152, 1:250; Santa Cruz Biotechnology, TX, USA). Western blot images were taken using Uvitec Cambridge Chemiluminescence Imaging System with the help of Alliance Software (ver. 16.06) and quantified by Uviband software (ver. 15.03) provided with the instrument (Uvitec Cambridge, Cambridge, UK).

### 2.4. Polysome Profiling

Polysome profiling was performed as previously described by [43]. Briefly, Huh7 cells were treated with 100 μg/mL cycloheximide (Sigma-Aldrich, Saint-Luis, MO, USA) for 10 min at 37 °C and washed twice with 100 μg/mL cycloheximide in 1xPBS. Afterwards, cells were scrapped using polysome extraction buffer (PEB) composed of 20 mM Tris HCl pH 7.5, 5 mM MgCl_2_, 100 mM KCl, 100 μg/mL cycloheximide, 0.5% Nonidet P-40 (Sigma-Aldrich), and 100 units’ Recombinant RNase Inhibitor (2313A, TAKARA, Otsu, Shiga, Japan), and then, the cell suspension was transferred to a 1.7-mL microfuge tube. The cell suspension was incubated on ice for 15 min with occasional inverting every 3 min (no vortex) and centrifuged at 12,000× *g* for 10 min at 4 °C in order to pellet the nuclei and debris. The cytoplasmic lysate (supernatant) was transferred to a fresh tube and protein concentration was measured by Bradford assay. Next, cytoplasmic cell lysates (1 mg of total protein) were layered upon a 10–50% sucrose gradient cushion and centrifuged at 190,000× *g* (SWTH64i rotor, Sorvall Ultra Pro80 Centrifuge, Thermo Fisher Sci., Waltham, MA, USA) for 90 min at 4 °C. After centrifugation, polypropylene tubes were pierced using a syringe needle and 12 fractions were collected (1 mL each). RNA absorbance in each fraction was monitored at 260 nm to record the polysome profile (Nanodrop 2000 Spectrophotometer, Thermo Fisher Sci.).

### 2.5. RNA Extraction, cDNA Preparation, and Quantitative PCR

Total RNA from Huh7 cells was isolated using the NucleoZOL reagent (Macherey-Nagel, Duren, Germany), and cDNA was synthesized with the High Capacity Reverse Transcription Kit (Applied Biosystems, Thermo Fisher Scientific Inc.). Real-time PCR was performed with SYBR^®^ Green (Applied Biosystems) in a LightCycler 96 apparatus (Roche Life Science, Basel, Switzerland). The mRNAs encoding *HIF1A*, *EPAS1*, *EPO*, *SERPINE1*, *PGK1*, *CYP24A1,* and *18S* were amplified using primers described in Table 1. Each sample was assayed in duplicate for both target and internal control. Relative quantitative gene expression was calculated using the ΔΔCT method and presented as fold increase in relation to the respective controls.

### 2.6. Cell Proliferation Assay

For cell proliferation assay, Huh7 cells were seeded into 96-well plates (2000 cells/well) and treated with 100 nM of 1,25(OH)_2_D3 restocked every 24 h for the indicated periods under 21% or 1% O_2_. Alternatively, Huh7 cells (2000 cells/well) were incubated with HIF-1α siRNA (10 nM; Qiagen, Venlo, Netherlands) in the presence of VIROMER^®^BLUE transfection reagent (Lipocalyx, Germany) according to the manufacturer’s instructions. Then, 24 h post-transfection, cells were incubated under 21% or 1% O_2_ for additional 24 or 48 h. Cell proliferation was measured using the “WST-1 Cell Proliferation Assay Kit” (TAKARA, MK400) in a Spark^®^ multiplate reader (Tecan, Männedorf, Switzerland) and values were normalized to those in the absence of cells for each condition.

### 2.7. Statistical Analysis

Statistical differences between two groups of data were assessed using the unpaired *t*-test in the GraphPad Prism version 5.04 software; *p* < 0.05 was considered to be significant (* *p* < 0.05; ** *p* < 0.01; *** *p* < 0.001).

## 3. Results

### 3.1. Calcitriol Reduces Both HIF-1α and HIF-2α Protein Levels and Hampers HIF-1 and HIF-2 Transcriptional Activity in Dose and Time Dependent Manner

**Figure 1:**
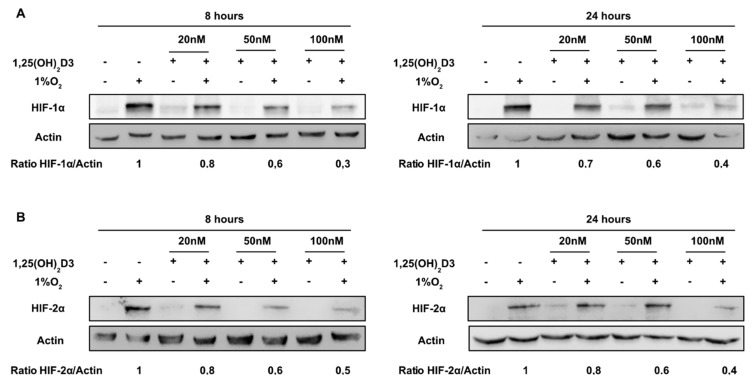
Calcitriol reduces hypoxia-inducible factors 1α (HIF-1α) and HIF-2α accumulation induced under hypoxia in a dose-dependent manner. Huh7 cells were treated with calcitriol (20, 50, or 100 nM) and incubated under hypoxia (1% O_2_) or normoxia (21% O_2_) for 8 or 24 h before collection and lysis. Cell lysates were analyzed for HIF-1α (**A**) and HIF-2α (**B**) protein expression by immunoblotting. The numbers below the panels represent the HIF-1α/actin or HIF-2α/actin levels average ratio, according to densitometric analysis of blots from three independent experiments.

In order to investigate the impact of calcitriol on HIF-1α and HIF-2α protein levels, Huh7 cells were incubated with increasing concentrations of 1,25(OH)_2_D3 (20, 50, and 100 nM) for 1 h before being subjected to normoxic (21% O_2_) or hypoxic (1% O_2_) conditions for 8 and 24 h. Total cell extracts were analyzed by Western immunoblotting, with antibodies against HIF-1α (Figure 1A) and HIF-2α (Figure 1B). Under normoxic conditions HIF-1α and HIF-2α basal protein levels are not significantly affected by calcitriol (Figure 1). However, calcitriol significantly suppresses induction of both HIF-1/2α subunits by hypoxia in a dose-dependent manner. Furthermore, Huh7 cells treated with calcitriol 1 h prior or after the hypoxic treatment, and for a total of 8 h, showed similar decrease in HIF-1/2α protein levels (Figure S1A).

**Figure 2:**
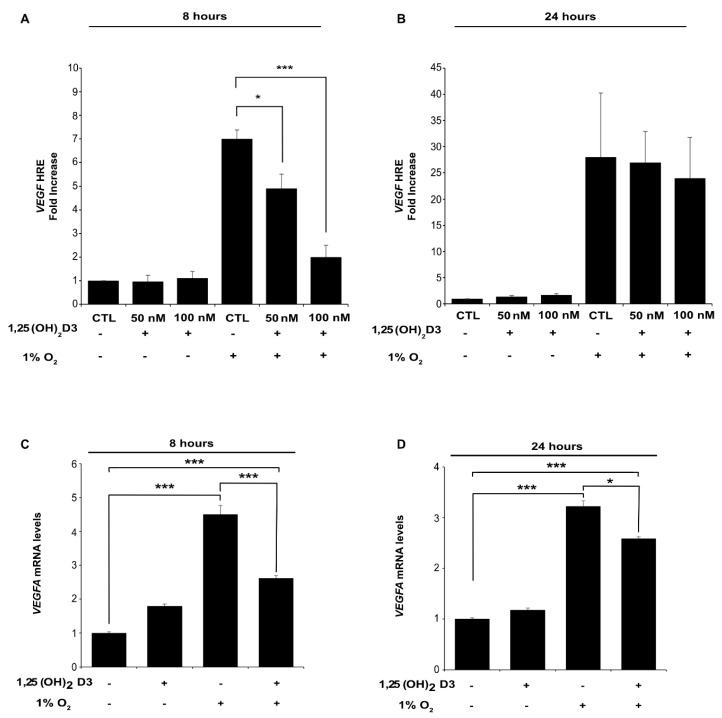
Calcitriol suppresses total HIF-1 and HIF-2 hypoxia-induced transcriptional activity in a dose-dependent manner. HIFs transcriptional activity was determined after transfection of Huh7 cells with the pGL3–5HRE-VEGF reporter plasmid (with the common for HIF-1 and HIF-2 promoter 5HRE-VEGF) and the control plasmid pCI-Renilla. Then, 24 h post-transfection, cells were treated with 1,25(OH)_2_D3 (50 nM or 100 nM) and incubated under hypoxia (1% O_2_) for (**A**) 8 h or (**B**) 24 h as indicated. Values show the fold increase in relative luciferase units (Firefly over Renilla activity) in relation to the values obtained from cells treated under normoxia 21% O_2_ in the absence of 1,25(OH)_2_D3 and represent the mean of three independent experiments performed in triplicate ± sem (* *p* < 0.05; *** *p* < 0.001). *VEGFA* mRNA levels were determined by quantitative real-time PCR in Huh7 cells treated with 100 nM of 1,25(OH)_2_D3 and incubated under hypoxia (1% O_2_) for (**C**) 8 h or (**D)** 24 h before collection. Results are shown as fold increase in relation to the values obtained from cells incubated under normoxia (21% O_2_) in the absence of 1,25(OH)_2_D3 and represent the mean ± sem of three independent experiments performed in triplicate (* *p* < 0.05; *** *p* < 0.001).

Subsequently, we wanted to examine whether the diminution of HIF-1/2α subunits protein levels by calcitriol also have negative impact on HIFs transcriptional activity. Therefore, we studied the effect of calcitriol on total HIFs activity by a reporter gene assay using the pGL3-5HRE-luciferase plasmid containing five copies of HREs from human VEGF (vascular endothelial growth factor) gene, which is a common HIF-1 and HIF-2 target gene (Figure 2A,B). Huh7 cells were transiently transfected with the reporter plasmids, and 24 h post-transfection, the cells were incubated with increasing concentrations of 1,25(OH)_2_D3 (50 and 100 nM) 1 h prior to their subsequent incubation to normoxia (21% O_2_) or hypoxia (1% O_2_). Exposure of the cells to hypoxic conditions for 8 h, induced HIF-1/2 activation by 7-fold compared to normoxia. Concurrent treatment with calcitriol, drastically inhibited HIFs transcriptional activity in a dose-dependent manner. In the presence of the 100 nM of the hormone, more than threefold suppression was observed (*p* < 0.001) (Figure 2A). Incubation of the cells under hypoxic conditions for 24 h induced HIF activation by 27-fold compared to normoxia, whereas concomitant treatment with 1,25(OH)_2_D3 did not lead to statistically significant inhibition of HIFs transcriptional activity, although luciferase values exhibited notable deviation (Figure 2B). Exposure of the cells to normoxic conditions for either 8 or 24 h did not cause detectable HIF transcriptional activation that was not altered by increasing doses of 1,25(OH)_2_D3 (Figure 2A,B). In parallel, Huh7 cells treated with 100 nM calcitriol for 8 or 24 h under normoxia (21% O_2_) or hypoxia (1% O_2_) exhibited significant decrease in *VEGFA* mRNA levels in both time periods when compared to hypoxically induced *VEGFA* mRNA (Figure 2C,D). To test whether this decrease in HIF-related transcriptional activity is also reflected to VEGF protein levels, cell lysates of Huh7 treated with calcitriol for 8 or 24 h were analyzed by Western blotting. Moreover, apart from hypoxic treatment, the prolyl-hydroxylase inhibitor DMOG (1 mM) was used to stabilize HIF-α subunits under normoxia (Figure S1B). Calcitriol treatment sharply reduced HIF-1α and HIF-2α levels irrespective of hypoxia or DMOG treatment. At the same time, ARNT level was also decreased by calcitriol albeit to a substantially lesser extent than HIF-α. Importantly, calcitriol was able to decrease VEGF protein levels only at hypoxic or DMOG-treated cells (more evident at 24 h) and not at normoxic cells indicating that calcitriol effect on VEGF levels is HIF dependent (Figure S1B). In order to verify our results in another established hepatocellular carcinoma cell line, HepG2 cells were transiently transfected with HRE and Renilla reporter plasmids and 24 h post-transfection, cells were incubated with 100 nM of 1,25(OH)_2_D_3_ 1 h prior to incubation in normoxia (21% O_2_) or hypoxia (1% O_2_) (Figure S1C,D). Calcitriol treatment for either 8 or 24 h significantly reduced HRE-related transcriptional activity corroborating results from Huh7 cells (Figure S1C). Furthermore, immunoblot analysis of the same HepG2 cell lysates revealed that calcitriol diminished HIF-1α (in both 8 and 24 h) and VEGF (after 24 h) levels (Figure S1D).

**Figure 3:**
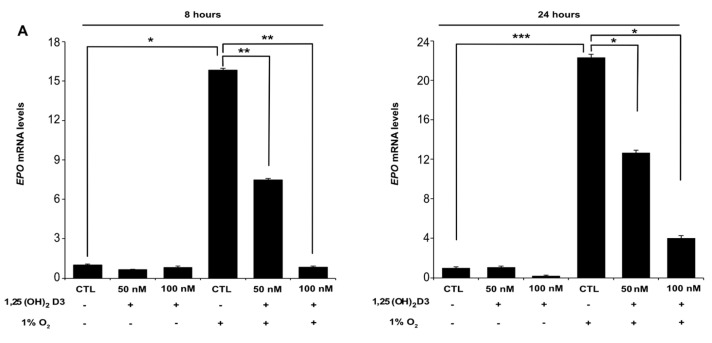

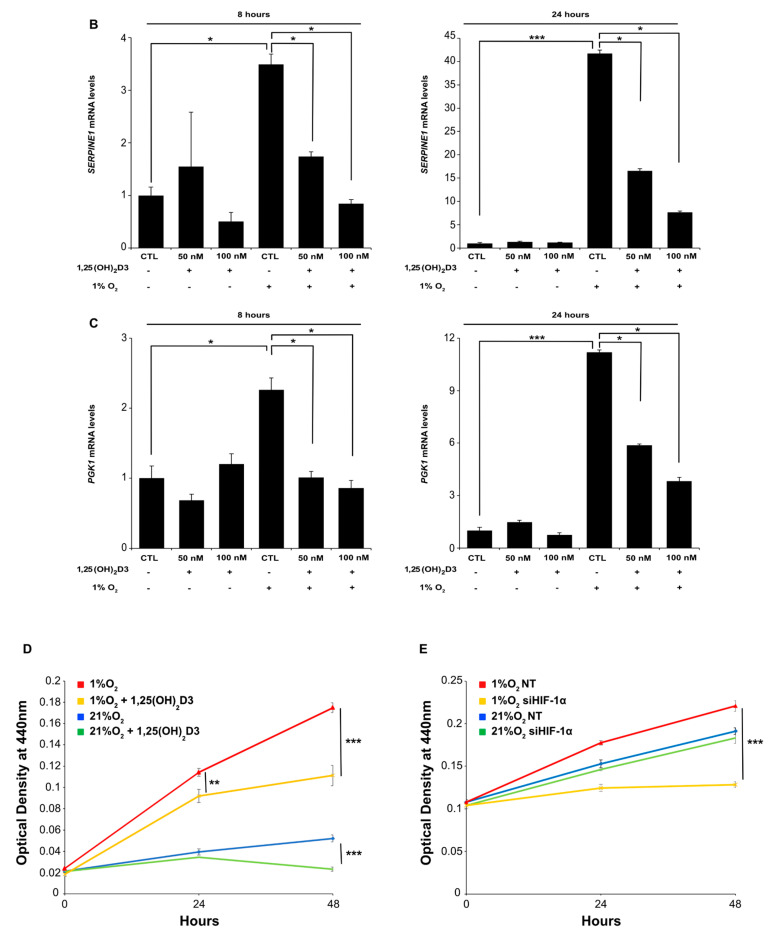
Calcitriol downregulates the expression of both HIF-2 and HIF-1 target genes and decreases Huh7 cells proliferation under hypoxia: *EPO* (**A**) and *SERPINE1* (**B**) are specific HIF-2 target genes, while **(C)** *PGK-1* is a specific target gene of HIF-1. mRNA levels of target genes were determined by quantitative real-time PCR in Huh7 cells treated with 1,25(OH)_2_D3 (50 or 100 nM) and incubated under hypoxia (1% O_2_) for 8 h (left panel) or 24 h (right panel) before collection. Results are shown as fold increase in relation to the values obtained from cells incubated under normoxia (21% O_2_) in the absence of 1,25(OH)_2_D3 and represent the mean ± sem of three independent experiments performed in duplicate (* *p* < 0.05; *** *p* < 0.001). (**D**,**E**) Determination of cell proliferation of Huh7 cells treated with 100 nM 1,25(OH)_2_D3 (**D**) or HIF-1α siRNA (**E**) and incubated under normoxia or hypoxia for the indicated time periods. Data are the mean from two independent experiments performed in quadruplets ± sem (n = 8; ** *p* < 0.01, *** *p* < 0.001).

Since both HIF-1α and HIF-2α are equally able to induce HRE-mediated transcription of the reporter plasmid, transcriptional activity levels measured by luciferase assays correspond to induction by both HIF-α isoforms. In order to evaluate the influence of 1,25(OH)_2_D3 on the transcriptional activity of each HIF-α isoform separately, we measured the mRNA levels of HIF-1 or HIF-2 specific target genes. Huh7 cells were incubated with increasing concentrations of 1,25(OH)_2_D3 (50 and 100 nM) for 1 h before being subjected to normoxic (21% O_2_) or hypoxic (1% O_2_) conditions for 8 and 24 h, and then, mRNA levels of erythropoietin (*EPO*) (Figure 3A), plasminogen activator inhibitor-1 (*SERPINE1*) (Figure 3B), and phosphoglycerate kinase-1 (*PGK1*) (Figure 3C) were estimated by quantitative RT-PCR. *EPO* and *SERPINE1* are specific targets of HIF-2 in Huh7 cells [24,30,44,45], while *PGK-1* is a HIF-1-specific target gene in the same cells [44,46]. In all cases, cells exposure to hypoxia for 8 or 24 h significantly elevated *EPO*, *SERPINE1,* and *PGK1* mRNA levels compared to normoxia. However, concomitant treatment of cells with increasing concentrations of 1,25(OH)_2_D3 significantly decreased *EPO*, *SERPINE1,* and *PGK1* mRNA levels in a dose-dependent manner (Figure 3). Under normoxia, the impact of calcitriol on the investigated genes was not significant.

Activation of HIFs is essential for cancer cells to adapt under low oxygen conditions. So, we wondered if calcitriol by reducing HIF-1/2α levels and activity could interfere with Huh7 cells ability to proliferate under hypoxia. To this end, Huh7 cells were kept under normoxia (21% O_2_) or hypoxia (1% O_2_) for a period of 48 h in the presence or not of 100 nM of calcitriol and their proliferation was determined by means of “WST-1 proliferation assay system.” Increased proliferation of Huh7 cells was observed in hypoxia when compared to normoxic conditions as it was previously described [47,48]. However, calcitriol-treated Huh7 cells exhibited significantly lowered proliferation rates at 24 and 48 h under hypoxia, whereas, under normoxia, calcitriol affected Huh7 proliferation to a much lesser extent and only after 48 h of treatment (Figure 3D). At the same time, silencing HIF-1α by siRNA greatly reduced Huh7 proliferation rates only under hypoxia (Figure 3E). These results indicate that calcitriol is able to interfere with HIF-dependent biological processes that enable cancer cells to thrive under hypoxic conditions. 

### 3.2. Negative Regulation of HIF-1/2α Protein Levels and HIFs Transcriptional Activity by Calcitriol is not Mediated by VDR Activation by Calcitriol

**Figure 4:**
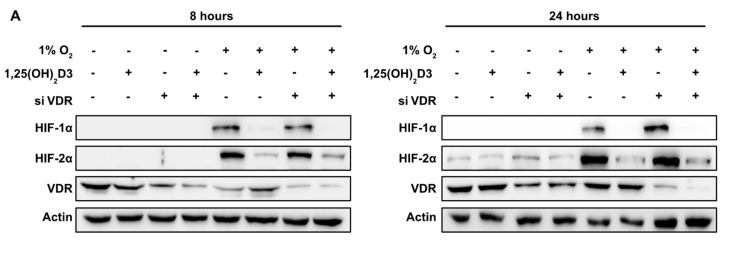

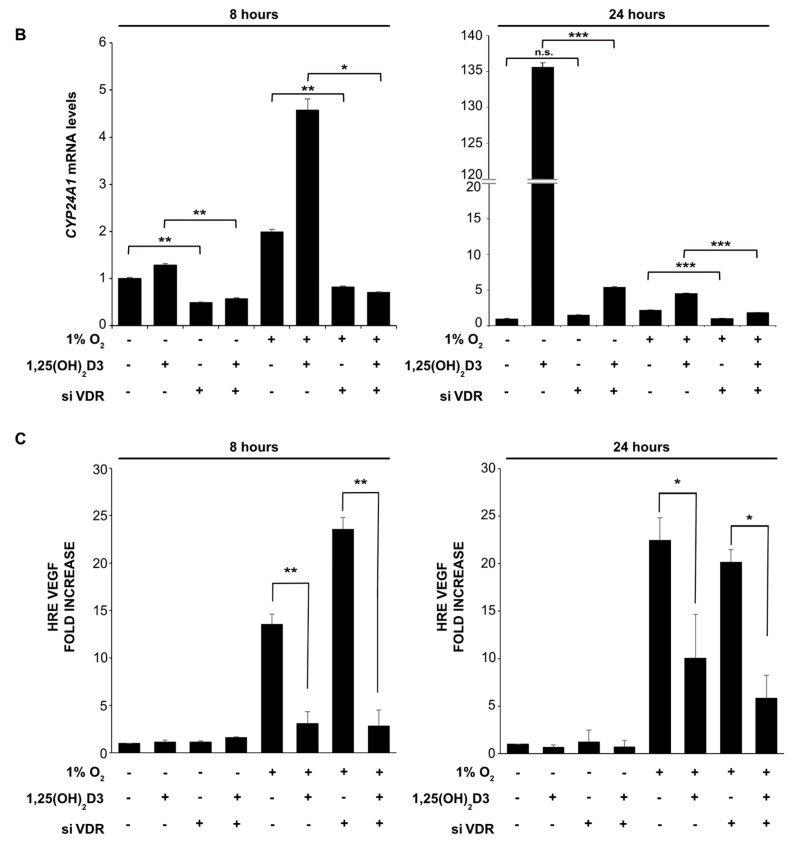
Suppression of vitamin D receptor (VDR) expression by siRNA does not abrogate 1,25(OH)2D3 negative regulation of HIF-1/2α protein levels and HIFs transcriptional activity. (**A**) Western blot analysis of HIF-1α, HIF-2α, and VDR protein levels in Huh7 lysates of cells incubated under normoxia or hypoxia in the presence (100 nM) or absence of calcitriol and impact of VDR siRNA on these levels. Actin is used as a loading control. (**B**) Impact of VDR silencing on CYP24A1 mRNA levels was determined in Huh7 cells treated with 1,25(OH)_2_D3 (100 nM) and incubated under hypoxia (1% O_2_) for 8 h (left panel) or 24 h (right panel) before collection. Results are shown as fold increase in relation to the values obtained from cells treated under normoxia 21% O_2_ in the absence of 1,25(OH)_2_D3 and represent the mean ± sem) of three independent experiments performed in duplicate (n.s. *p* > 0.05, * *p* < 0.05; ** *p* < 0.01; *** *p* < 0.001). (**C**) Determination of HIFs transcriptional activity after transfecting Huh7 cells with control or VDR siRNA, the pGL3–5HRE-VEGF reporter plasmid and the plasmid pCI-Renilla. Then, 24 h post-transfection, cells were treated with 1,25(OH)_2_D3 (100 nM) and incubated under hypoxia (1% O_2_) for 8 h (left panel) or 24 h (right panel) as indicated. Values show the fold increase in relative luciferase units (Firefly over Renilla activity) in relation to the values obtained from cells transfected with the control siRNA under normoxia 21% O_2_ in the absence of 1,25(OH)_2_D3 and represent the mean of three independent experiments performed in triplicate ± sem) (* *p* < 0.05; ** *p* < 0.01).

In order to investigate whether the observed effects of 1,25(OH)_2_D3 on HIFs are dependent on VDR activation by calcitriol, we performed VDR silencing experiments. Huh7 cells were transfected with siRNA-VDR (or control siRNA). Then, 48 h after transfection, cells were incubated with 1,25(OH)_2_D3 (100 nM) for 1 h before being subjected to normoxic (21% O_2_) or hypoxic (1% O_2_) conditions for 8 and 24 h. Total cell extracts were analyzed by Western immunoblotting, with antibodies against HIF-1α and HIF-2α. VDR protein levels were estimated for the assessment of silencing efficiency and more than 50% reduction in VDR protein levels was detected (Figure 4A). In order to ensure that the observed reduction in VDR protein levels is adequate to abrogate its genomic function, and to further support our hypothesis, we investigated the expression of CYP24A1 gene, a well-established target gene of calcitriol-activated VDR that encodes the catabolizing calcitriol, in negative feedback mode, enzyme CYP24A1 [49,50]. The CYP24A1 mRNA levels were measured by quantitative real-time PCR with and without silencing of VDR in the presence or absence of calcitriol (Figure 4B). Indeed, calcitriol induced the transcription of CYP24A1 in VDR-depended manner and silencing of VDR with reported above efficiency abrogates CYP24A1 gene induction and thus demonstrates successful nullification of VDR transcriptional potential. At the same time, VDR silencing does not reverse calcitriol’s negative impact on HIF-1/2α protein levels (in both siRNA-treated and control cells) (Figure 4A) and HIF-mediated transcriptional activity (Figure 4C) suggesting a VDR-independent regulation by calcitriol.

Exposure of cells to hypoxic conditions for 8 h, induced HIFs activation by 12-fold compared to normoxia. In accordance with our previous results, concomitant treatment with calcitriol, led to significant (*p* < 0.01) suppression of HIFs transcriptional activity (Figure 4C). Incubation of cells under hypoxic conditions for 24 h induced HIF activation by 23-fold compared to normoxia, whereas concomitant treatment with 1,25(OH)_2_D3 led to statistically significant inhibition of HIF transcriptional activity. At both time points, silencing of VDR does not abrogate the negative regulation of HIFs’ activity by calcitriol (Figure 4C). Interestingly, VDR silencing alone (only in calcitriol-untreated cells) could modestly increase HIF-1/2α levels (Figure 4A; 24 h) and activity (Figure 4C; 8 h). Altogether, our results demonstrate that 1,25(OH)_2_D3 suppresses HIF-1α and ΗIF-2α protein expression and HIF-1/2 transcriptional activity in a VDR-independent nongenomic mechanism.

### 3.3. Calcitriol Suppresses HIF1A and EPAS1 mRNA Translation by Downregulating Components of PI3K/Akt/mTOR Signaling Pathway

**Figure 5:**
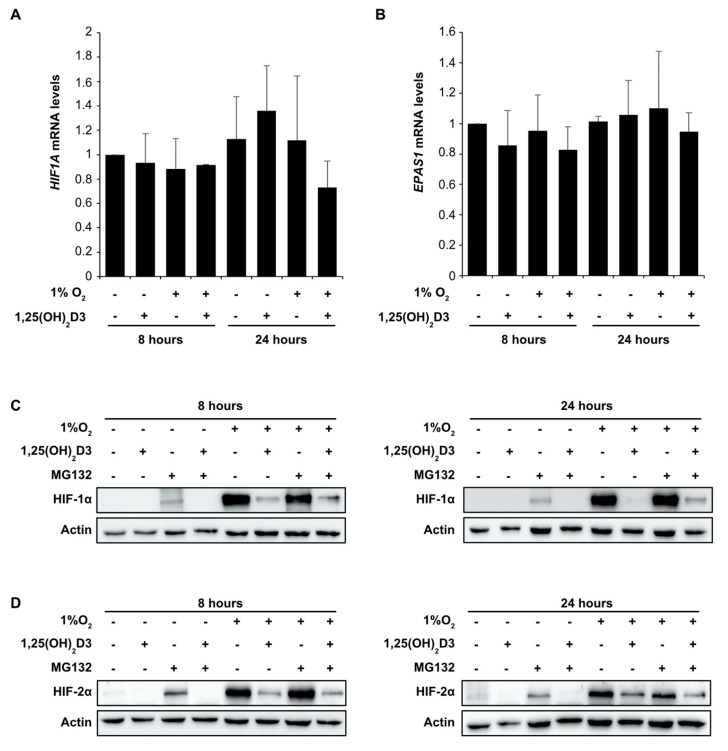
Calcitriol does not affect *HIF1A* and *EPAS1* mRNA levels or proteasomal degradation of HIF-1/2α subunits. Determination of (**A**) *HIF1A* and (**B**) *EPAS1* mRNA levels by quantitative real-time PCR in Huh7 cells treated with 1,25(OH)_2_D3 (100 nM) and incubated under hypoxia (1% O_2_) for 8 or 24 h before collection. Results are shown as fold increase in relation to the values obtained from cells treated under normoxia 21% O_2_ in the absence of 1,25(OH)_2_D3 and represent the mean ± sem) of two independent experiments performed in duplicates. Western blot analysis of (**C**) HIF-1α and (**D**) HIF-2α protein levels in Huh7 cell lysates. Cells were treated with 1,25(OH)_2_D3 (100 nM) and incubated under hypoxia (1% O_2_) for 8 h (left panel) or 24 h (right panel). Cells were concomitantly treated with 10 μΜ MG132 for 4 h before collection and lysis. Actin is used as a loading control.

Since VDR is not implicated in the downregulation of HIF by calcitriol, we examined the effects of 1,25(OH)_2_D3 on HIF-1α and HIF-2α at transcriptional and posttranscriptional level. Initially, we measured *HIF1A* (Figure 5A) and *EPAS1* (Figure 5B) mRNA levels from Huh7 cells treated with 1,25(OH)_2_D3 (100 nM) under hypoxia (1% O_2_) for 8 and 24 h. These experiments revealed that both *HIF1A* and *EPAS1* mRNA levels were not significantly (*p* > 0.05) altered by 1,25(OH)_2_D3 at 8 or 24 h (Figure 5A,B) implying that calcitriol does not affect *HIF1A* and *EPAS1* genes transcription.

At posttranscriptional level, the concentration of HIF-1/2α subunits is controlled by the rate of mRNA translation in relation to their proteasomal degradation [23,27]. Therefore, we initially investigated whether calcitriol influences HIF-1/2α subunits proteasomal degradation. For that reason, Huh7 cells were incubated with 1,25(OH)_2_D3 (100 nM) for 1 h before being subjected to normoxic (21% O_2_) or hypoxic (1% O_2_) conditions for 8 and 24 h. To attain proteasomal inhibition, cells were concurrently treated with 10 μΜ of proteasome inhibitor MG132 for 4 h before lysis. Total cell extracts were analyzed by immunoblotting, with antibodies against HIF-1α (Figure 5C) and HIF-2α (Figure 5D). Proteasomal inhibition by MG132 leads to HIF-1α and HIF-2α accumulation under normoxic conditions as expected. However, HIF-1α and HIF-2α protein levels remain undetectable after concomitant treatment with calcitriol (Figure 5C,D). Under hypoxic conditions, calcitriol significantly suppresses accretion of both HIF-1/2α subunits by hypoxia in agreement with our previous results. However, the suppression of HIF-1α and HIF-2α protein levels by calcitriol is not abrogated by use of MG132 inhibitor (Figure 5C,D) and this indicate that 1,25(OH)_2_D3 does exert its effect via the proteasomal degradation of HIF-1/2α subunits.

**Figure 6:**
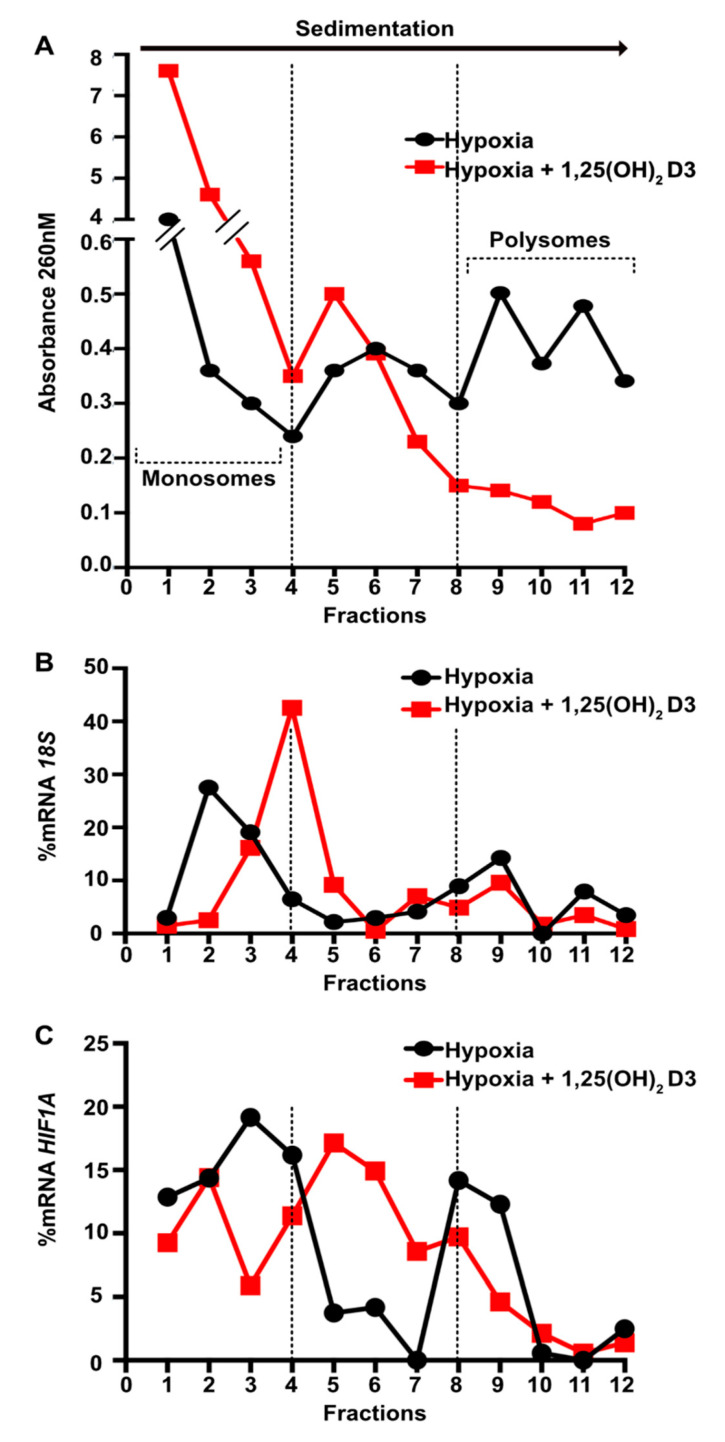

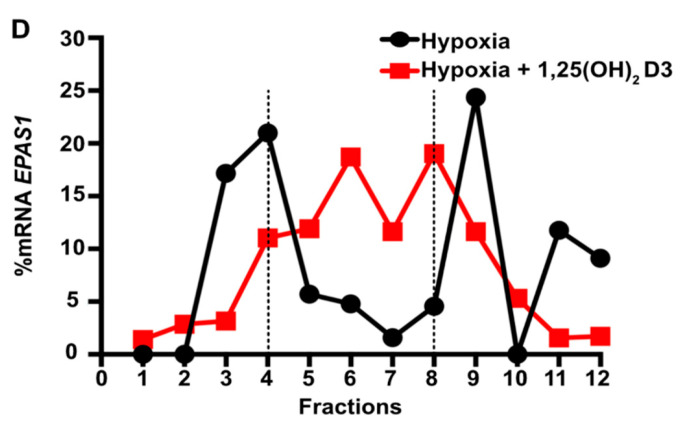
Calcitriol inhibits *HIF1A* and *EPAS1* mRNA translation to protein. Huh7 cells treated with 1,25(OH)_2_D3 (100 nM) and incubated under hypoxia (1% O_2_) for 8 h. Cells were treated with cycloheximide and lysed and fractionated on a 10–50% sucrose gradient cushion. (**A**) Representative λ = A_260_ nm fractionation profiles. (**B**) Distribution of 18S rRNA determined by qRT-PCR. (**C**) *HIF1A* mRNA distribution between fractions as indicated. (**D**) *EPAS1* mRNA distribution between fractions as indicated. Fraction 1 corresponds to the top of the gradient (free mRNAs) and 12 corresponds to the bottom of the gradient. Translated mRNAs are associated with the heavy polysomal fractions (from fraction 8 to 12; as indicated). Graphs depict representative data and are expressed as a percentage of total RNA of interest in the gradient from two independent experiments.

As it is mentioned above, the accumulation of HIF-1/2α subunits is the result of a subtle balance between protein synthesis and their degradation, so, we next examined the effect of 1,25(OH)_2_D3 on the rate of *HIF1A* and *EPAS1* mRNA translation to protein. To assess whether calcitriol regulates HIF-α subunits at the level of mRNA translation, we characterized *HIF1A* and *EPAS1* mRNA polysomal association in cytoplasmic extracts of Huh7 cells (Figure 6). Huh7 cells were incubated with 1,25(OH)_2_D3 (100 nM) for 1 h before being subjected to hypoxia (1% O_2_) for 8 h. The first fraction (#1) corresponds to the upper layer of the gradient that contains free mRNAs and the last fraction (#12) corresponds to the lowest layer of the gradient that contains heavy polysomes. Translated mRNAs are associated with the heavy polysomal fractions 8 to 12, as indicated by the presence of 18S rRNA (Figure 6B). A_260_ profiles of gradient fractions were obtained from cells subjected to hypoxia in the absence or presence of 1,25(OH)_2_D3 (Figure 6A). We observe a strong RNA accumulation in monosomes with a concurrent reduction in polysomal fractions in cells treated with 1,25(OH)_2_D3 (Figure 6A). Subsequently, we analyzed the distribution of *HIF1A* and *EPAS1* mRNAs along the gradient. In the absence of 1,25(OH)_2_D3, the mRNAs coding for HIF-1α and HIF-2α were equally distributed in both monosome and polysome fractions, suggesting that a large amount of these mRNAs is translated during hypoxia. After treatment with calcitriol, *HIF1A* and *EPAS1* mRNAs concentrated in the middle fractions of the gradient that contain ribosomes with low translation rates (Figure 6C,D). Altogether, these results demonstrate that calcitriol abrogates the recruitment of *HIF1A* and *EPAS1* mRNAs into active polysomes under hypoxia and thus suppresses *HIF1A* and *EPAS1* mRNA translation.

**Figure 7:**
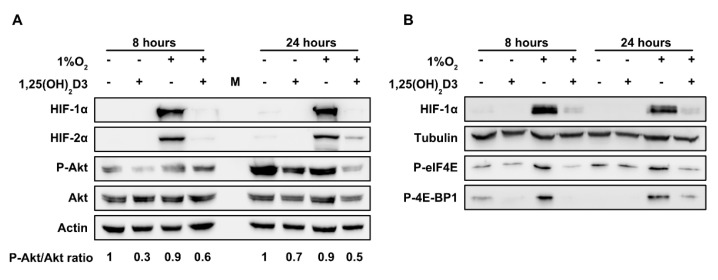
Calcitriol suppresses HIF-1/2α subunits protein synthesis at the level of *HIF1A* and *EPAS1* mRNA translation by downregulating components of the PI3K/Akt/mTOR signaling pathway. Western blot analysis of (**A**) phosphorylated Akt and total Akt (numbers below blots represent P-AKT/AKT ratios from three independent experiments. In each case, ratio value of 1 is given to normoxic conditions in the absence of calcitriol). (**B**) phosphorylated eIF4E and phosphorylated 4E-BP1 protein levels in Huh7 cell lysates. Cells were treated with 1,25(OH)_2_D3 (100 nM) and incubated under hypoxia (1% O_2_) for 8 or 24 h as indicated. Actin is used as a loading control for Figure 7A. Tubulin is used as a loading control for Figure 7B.

Previous studies have shown that the PI3K/Akt/mTOR signaling pathway plays a key role in the control of *HIF1A* and *EPAS1* mRNA translation to protein [27]. In order to explore whether the inhibition of HIF-1/2α subunits’ protein synthesis by calcitriol is linked to the downregulation of this pathway, Huh7 cells were pretreated for 1 h with 1,25(OH)_2_D3 (100 nM) and incubated under hypoxia (1% O_2_) for 8 and 24 h. Thereafter, the levels of phosphorylated or total Akt and of its downstream targets eIF4E and 4E-BP1 were examined by Western blot. HIF-1α and HIF-2α were used as a control for hypoxic treatment. Both HIF-1α and HIF-2α were accumulated after 8 or 24 h of hypoxic treatment and, as expected, were suppressed by calcitriol at each time point. The levels of phosphorylated Akt were modestly suppressed by calcitriol after 8- and 24-h incubation under hypoxia. In normoxia, there is also a decrease in phospho-AKT levels by calcitriol after 8 and 24 h of treatment (Figure 7A). At the same time, the levels of nonphosphorylated Akt were not altered. Moreover, the levels of phosphorylated eIF4E and 4E-BP1 were dramatically reduced by calcitriol after 8 or 24 h of hypoxic treatment (Figure 7B), with minimal variations under normoxia, indicating the downregulation of Akt pathway by calcitriol.

## 4. Discussion

In order to adapt and survive in the poorly oxygenated environment of hypoxic solid tumors, cancer cells heavily depend on the activation of HIFs. The overexpression of oxygen-regulated HIF-α subunits is frequently encountered and associated with cancer pathogenesis, poor patient prognosis, and resistance to conventional therapies [51]. As a consequence, there is a rising interest for discovering potent inhibitors of HIF-mediated signaling (naturally occurring or synthetic) in order to be tested along with more traditional approaches for the treatment of different types of cancer [52,53]. HIFs affect important cancer hallmarks including cell proliferation, apoptosis, differentiation, vascularization/angiogenesis, genetic instability, tumor metabolism, tumor immune responses, and invasion and metastasis [54]. At the same time, accumulating data suggest that vitamin D can modulate the entire process of tumorigenesis, from initiation to metastasis by regulation of the same hallmarks (cell proliferation, differentiation, apoptosis, and angiogenesis) and thus cross-talking between HIF-mediated signaling and vitamin D can be supposed [3,7,15,55,56]. Up to now, there is little knowledge on the effects of calcitriol on HIF-1 and HIF-2 and on how calcitriol exerts these effects. The inhibition of HIF-1α protein expression and HIF-1 transcriptional activity by calcitriol was previously demonstrated in a number of cancer cells [57]. However, the mechanism of calcitriol action was not investigated as well as the impact of calcitriol on HIF-2α and HIF-2, and thus, it was not clear if calcitriol is pan-HIFs-α and HIFs inhibitor. This is important since the inhibition of one HIFα subunit, e.g., HIF-1α, induces the over-expression of HIF-2α, and thus, inhibitors targeting simultaneously HIF-1 and HIF-2 may be more effective in the therapy of some cancer types [58]. Therefore, clarifying the mechanisms of vitamin D action in cancer will contribute to the development of efficient strategies of vitamin D and its analogs application in cancer therapy and prevention.

Here, we provide the evidence that calcitriol impairs the transcriptional activity of both HIF-1 and HIF-2 isoforms mainly by diminishing HIF-1/2α protein levels (Figure 1, Figure 2, Figure 3). However, the downregulation of HIF-1/2α subunits in the presence of calcitriol is not related to decreased transcription of *HIF1A* or *EPAS1*, or the proline hydroxylation pathway that leads to proteasomal degradation of HIF-1/2α (Figure 5 and Figure S1B). Moreover, in a recent report, it was demonstrated that calcitriol does not alter PHD1/2 protein levels in oral epithelial cells [59]. Although apart from the canonical HIF-α degradation road, there are VHL-independent [28] and lysosomal degradation pathways [60] and their crosstalk with calcitriol remains to be tested. Additionally, analysis of *HIF1A* and *EPAS1* mRNAs distribution in polysome profiling experiments revealed that in the absence of 1,25(OH)_2_D_3_, the mRNAs coding for HIF-1α and HIF-2α were equally distributed in both monosome and polysome fractions, suggesting that a large amount of these mRNAs is translated during hypoxia. Treatment with calcitriol under hypoxia displaces *HIF1A* and *EPAS1* mRNA to ribosomal fractions with low translational capacity (Figure 6) and therefore inhibits *HIF1A* and *EPAS1* mRNAs translation to proteins. There are converging pathways that control components of the translational machinery and include (i) Pim2-mediated phosphorylation [61] and (ii) the ERK1/2 pathway, which mainly directly activates HIF-1/2α [30,31,32,62] but is activated by calcitriol (Figure S1E and [63]). Besides, the PI3K/Akt/mTOR pathway is known to be responsible for regulating the translation of HIF-1/2α subunits of mRNA [26,27]. However, there are conflicting data concerning the regulation of this pathway by calcitriol, and the existing reports claim both induction [9] as well as inhibition of PI3K/Akt/mTOR pathway components [64]. A recent study in liver cancer cells HepG2 revealed that 1,25(OH)_2_D3 inhibits cell proliferation by activating PTEN phosphatase and subsequent inhibition of the PI3K/Akt/mTOR pathway [65], in agreement with our results showing that calcitriol implicates HIF-mediated downregulation of Huh7 proliferation under hypoxia (Figure 3D,E).

Our data support that calcitriol has inhibitory effect on PI3K/Akt/mTOR signaling and, in particular, downregulates the phosphorylation of Akt in Huh7 cells as well as subsequent targets of Akt signaling proteins such as eIF4E and 4E-BP1 (Figure 7). The levels of phosphorylated eIF4E and 4E-BP1 are dramatically suppressed by calcitriol after 8 or 24 h of hypoxic treatment, further supporting that calcitriol suppresses HIF-1/2α subunits synthesis at the level of mRNA translation (Figure 6 and Figure 7B). Phosphorylation of eIF4E and 4E-BP1 is a crucial step for the initiation of cap-dependent protein translation [66,67].

The next question investigated was whether the observed calcitriol effects are carried out through genomic or nongenomic signaling. The majority of calcitriol biological effects described are genomic and mediated by VDR, which mainly acts by regulating the expression of genes that contain vitamin D response elements (VDREs) in the DNA sequence of their promoter [9,68]. The nongenomic action of calcitriol with rapid cellular response is substantially less investigated. In our experiments, silencing of VDR by siRNAs does not alter or abrogate negative regulation of HIF-1α and HIF-2α protein levels and HIF-1/2 transcriptional activity by calcitriol (Figure 4), therefore demonstrating that observed effect is VDR independent and follows nongenomic mechanism. Moreover, VDR silencing in calcitriol untreated cells either did not affect or increased HIF-1/2α levels and activity (Figure 4A,C), in agreement with a previous observation in a VDR knockout model [69]. The VDR silencing was confirmed by substantial reduction in VDR protein levels as well as by complete suppression of VDR target gene *CYP24A1* expression (Figure 4). Nowadays, it is accepted that nongenomic mechanism of calcitriol action is mediated by its binding to membrane vitamin D receptor named “MARRS or mVDR (membrane VDR)” and activation of intracellular signaling pathways [9].

Interestingly, we observe that silencing of VDR in the absence of calcitriol induces the transcriptional activity of HIF-1 and HIF-2. This may indicate the existence of an additional regulatory mechanism of HIF-1/2 by VDR alone that does not require the binding of the calcitriol to VDR. One hypothesis could be that VDR in the absence of its ligand is bound either in the HIF genes promoter or on HIF target genes promoters. It has also been suggested that VDR, by creating a complex with the IKKβ protein, leads to the deactivation of NF-κB, which has already been reported to transcriptionally activate HIF-1 [70,71,72]. Thus, the increase in HIF transcriptional activity after VDR silencing (in the absence of calcitriol) could be explained by this mechanism but needs further investigation.

**Figure 8:**
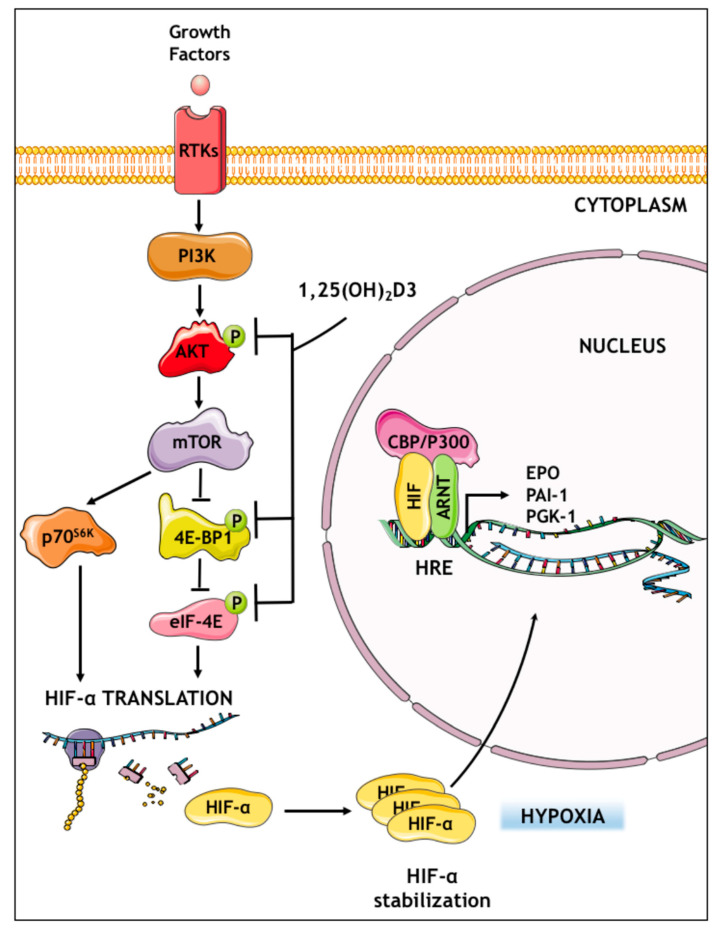
Suggested model of HIF-1/2 negative regulation by calcitriol in hypoxic Huh7 cells. Briefly, 1,25(ΟΗ)_2_D3 inhibits the activation of PI3K/AKT/mTOR pathway and, thus, impairs the phosphorylation of critical components of the translational machinery responsible for *HIF1A* and *EPAS1* mRNA translation. As a result, in the presence of calcitriol, HIF-1/2α subunits are not efficiently synthesized and HIF-1/2 activity is diminished.

Overall, the acquired data demonstrate that that calcitriol significantly reduces HIF-1α and HIF-2α protein levels and subsequent HIF transcriptional activity in Huh7 and HepG2 hepatocarcinoma cell lines selected as a model system for the investigation of calcitriol impact on HIF-1 and HIF-2. Hepatocellular carcinoma (HCC) is the most common type of primary liver cancer in adults. It is characterized by increased resistance to drugs that leads to high mortality rates. Although the primary molecular event of hepatocellular carcinogenesis remains unknown, the molecular pathogenesis of HCC is influenced by multiple factors. There are well-established events that contribute to malignant transformation of HCC such as the upregulation of RAS/RAF/MAPK and PI3K/AKT/mTOR signaling pathways, which play a prominent role in proliferation, survival, invasion, and metastasis of HCC cells [73,74]. These processes are related with neoangiogenesis and abnormal vessel formation, which, in turn, heavily rely on the development of a hypoxic microenvironment and stimulation of VEGF expression [75,76]. Summarizing, our results propose a model (Figure 8) by which calcitriol impairs the activation of the translational machinery downstream of PI3K/AKT pathway and, thus, decreases HIF-1/2α protein synthesis and HIF-mediated transcriptional activity in a VDR-independent manner.

## 5. Conclusions

HIF-1 and HIF-2 are the core components of the main signaling pathway that enables cancer cells to adapt and survive in the poorly oxygenated microenvironment of solid tumors. Here, we demonstrate that calcitriol is a potent HIF-1 and HIF-2 negative regulator and elucidate the mechanism of this effect (Figure 8). The experimental data support that calcitriol exerts its activity in a VDR-independent manner by downregulating components of the PI3K/Akt/mTOR signaling pathway responsible for the efficient translation of *HIF1A* and *EPAS1* mRNA and HIF-1/2α protein synthesis. Reduction in HIF-1α and HIF-2α proteins at the level of protein translation humpers HIF1/2 transcriptional activity. The presented data offer new insight on the mechanism of vitamin D anticancer action and, given the fact that both HIF-1 and HIF-2 are validated targets in cancer treatment and prevention, can contribute to the development of the efficient strategies in vitamin D-based cancer therapy.

## Figures and Tables

**Table 1:** Primer sequences used for qPCR.

Gene	Sequence (5′-3′)
*VEGFA*	F: CCCACTGAGGAGTCCAACATC R: GGCCTTGGTGAGGTTTGATC
*HIF1A*	F: TTTTTCAAGCAGTAGGAATTGGA R: GTGATGTAGTAGCTGCATGATCG
*EPAS1*	F: TTCCTATTCACCAAGCTAAAGGAG R: GACTCCTCGAAGTTCTGATTCC
*EPO*	F: AGGCCGAGAATATCAGACG R: CCATCCTCTTCCAGGCATAGAAA
*SERPINE1*	F: CAGCTGACAGGAGGAGA R: CCCATGAGCTCCTTGTACAGAT
*PGK1*	F: CTGTGGCTTCTGGCATACCT R: CGAGTGACAGCCTCAGTATA
*CYP24A1*	F: TCTCAAGAAACAGCACGACACCC R: GCACCGACTCAAAGGAACCCAAC
*18S*	F: CTCAACACGGGAAACCTCAC R: CGCTCCACCAACTAAGAACG

## Supplementary Materials

The following are available online at https://www.mdpi.com/2073-4409/9/11/2440/s1, Figure S1.
