# Supplementary material for: Calcitriol Suppresses HIF-1 and HIF-2 Transcriptional Activity by Reducing HIF-1/2α Protein Levels via a VDR-Independent Mechanism
Source: Cells. 2020 Nov 9;9(11):2440. doi: 10.3390/cells9112440 (PMC7695316; doi:10.3390/cells9112440)
Supplement: Supplementary file 1 [file cells-09-02440-s001.pdf]

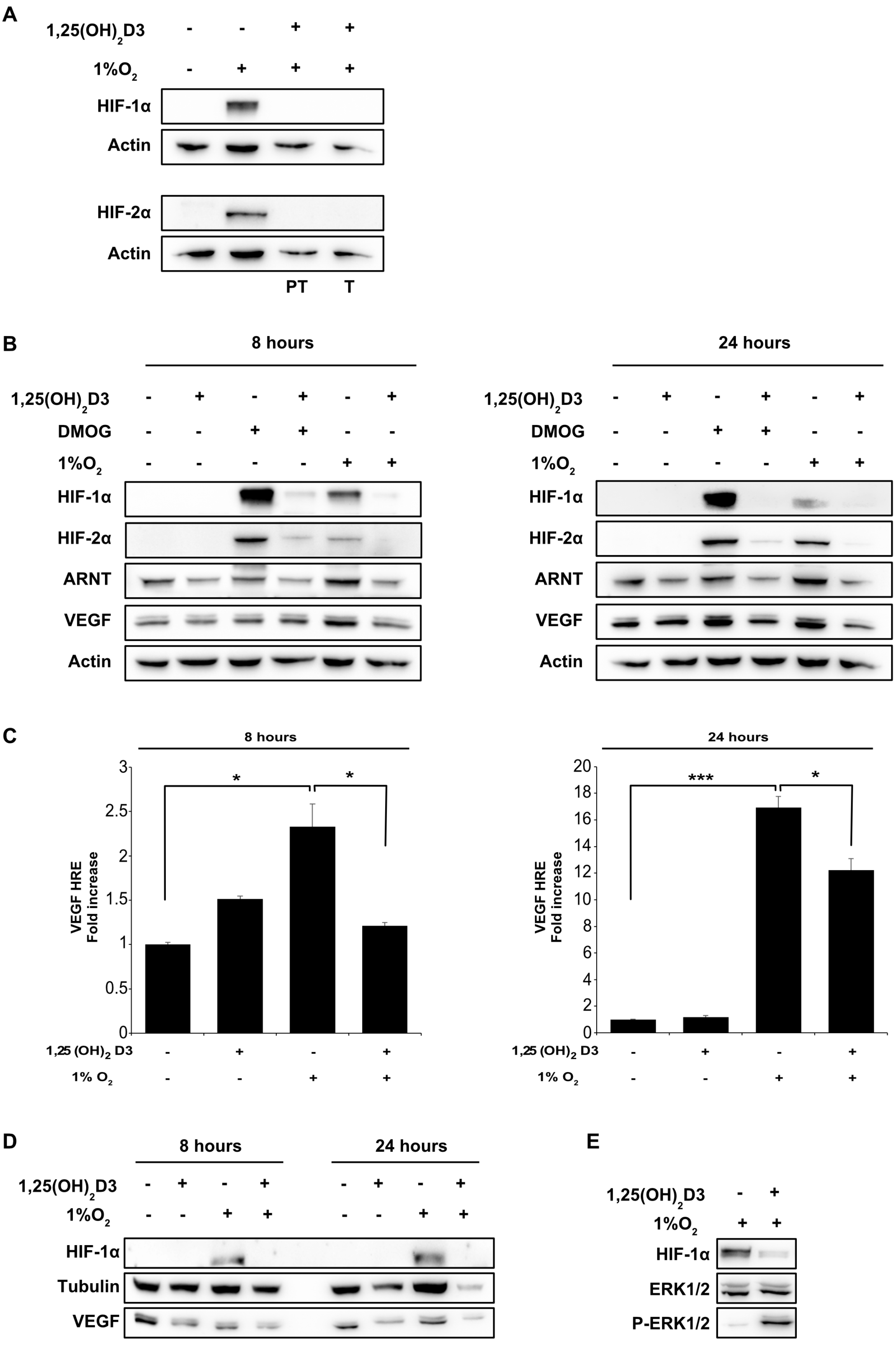

Figure S1: **(A)** Western blot analysis of HIF-1α and HIF-2α protein levels in Huh7 cell lysates. Cells were incubated under hypoxia (1% O_2) for 8 hours. Addition of 1,25 $(OH)_2D3$ (100nM) was made either 1 hour

prior (PT) or after (T) cells exposure to hypoxia. **(B)** Western blot analysis of HIF-1α, HIF-2α, ARNT and VEGF protein levels in Huh7 cell lysates. Cells were treated with 1,25 $(OH)_2D3$ (100nM) and incubated under hypoxia (1% O_2) for 8 hours (left panel) or 24 hours (right panel), or treated with 1mM DMOG and 1,25 $(OH)_2D3$ (100nM) for 8 hours (left panel) or 24 hours (right panel) before collection and lysis. Actin is used as a loading control. **(C)** HIFs transcriptional activity, determined after transfection of HepG2 cells with the pGL3–5HRE-VEGF reporter plasmid and the control plasmid pCI-Renilla. 24 hours post-transfection, HepG2 cells were treated with 1,25 $(OH)_2D3$ (100nM) and incubated under hypoxia (1% O_2) for 8 or 24 hours as indicated. Values show the fold increase of relative luciferase units (firefly over Renilla activity) in relation to the values obtained from cells treated under normoxia 21%O_2 in the absence of 1,25 $(OH)_2D3$ and represent the mean of two independent experiments performed in triplicate (± s.e.m) (*$P<0.05$; ***$P<0.001$). **(D)** Western blot analysis of HIF-1α and VEGF protein levels in HepG2 cell lysates. Cells were incubated with 1,25 $(OH)_2D3$ (100nM) and treated under hypoxia (1% O_2) for 8 or 24 hours as indicated. Tubulin is used as a loading control. **(E)** Western blot analysis of HIF-1α, total ERK1/2 and phosphorylated ERK1/2 protein levels in Huh7 cell lysates. Cells were incubated with 1,25 $(OH)_2D3$ (100nM) and treated under hypoxia (1% O_2) for 24 hours as indicated.
